# Unisensory and Multisensory Processing in the Integration of Bodily Information: An EEG Study Using the Rubber Hand Illusion

**DOI:** 10.1111/psyp.70368

**Published:** 2026-07-28

**Authors:** A. J. Sutil‐Jiménez, G. Alba, S. Duschek, M. A. Muñoz

**Affiliations:** ^1^ Department of Personality Assessment and Treatment at University of Granada (UGR) Granada Spain; ^2^ UMIT‐Institute of Psychology University for Health SciencesMedical Informatics and Technology Hall in Tirol Austria; ^3^ Mind, Brain and Behavior Research Center, at University of Granada (CIMCYC‐UGR) Granada Spain; ^4^ Department of Psychobiology & Behavioral Sciences Methods Complutense University of Madrid Pozuelo de Alarcón Spain

**Keywords:** alpha activity, beta activity, EEG, functional connectivity, multisensory integration, rubber hand illusion

## Abstract

Multisensory integration (MSI) is crucial to interact adaptively with our environment and create a coherent representation of the body. Body illusion techniques, such as the rubber hand illusion, provide useful tools to study MSI since they produce a conflict between different sensory modalities and alter body perception. While previous research revealed that body illusions modulate neural activity in unisensory and multisensory cortical areas during multisensory stimulation, cortical activity during unisensory stimulation has barely been studied. The present EEG study investigated brain mechanisms involved in MSI using unisensory (visual, tactile) and multisensory (visuotactile) stimulation. Alpha‐ and beta‐band power, as well as functional connectivity, were assessed in 36 subjects during visual, tactile, and visuotactile stimulation in the rubber hand illusion paradigm and a control condition. The illusion was associated with widespread reductions in alpha and beta power, alongside increased beta‐band connectivity between unisensory and multisensory cortical regions. Notably, alpha‐band effects revealed cross‐modal influences: visual stimulation modulated activity in somatosensory regions, while tactile stimulation affected occipital areas. Unisensory stimulation also elicited beta connectivity changes across modalities, with visual input enhancing sensorimotor coupling and tactile input increasing connectivity involving visual regions. The subjective strength of the illusion was associated with beta power reductions during visual stimulation, linking neural modulation to individual perceptual experience. These findings demonstrate that the illusory state is characterized by a global reconfiguration of sensory processing, extending beyond multisensory contexts and shaping unisensory processing through cross‐modal interactions.

## Impact Statement

This study demonstrates that body illusions not only alter multisensory processing but also reshape unisensory visual and tactile processing. Using EEG oscillatory activity and connectivity, we show that the rubber hand illusion reduces differences between unisensory and multisensory stimulation, suggesting cross‐modal adjustments in sensory processing. These findings advance current knowledge by highlighting unisensory contributions to sensory conflict resolution and the neural mechanisms of multisensory integration.

## 1 Introduction

The integration of stimuli from multiple sensory modalities is crucial to interact adaptively with our environment and maintain awareness of our own body. Research into multisensory integration (MSI) has shown that when visual information from an external object is incongruent with tactile information from one's own body, the brain may resolve this conflict by creating a body illusion (Botvinick and Cohen 1998; Guterstam et al. 2013; Litwin 2020). During such an illusion, the external object is perceived as part of one's own body, while somatosensory accuracy decreases (Dempsey‐Jones and Kritikos 2014; Faivre et al. 2017; Kanayama et al. 2016; Özkan et al. 2023; Riemer et al. 2019; Shibuya et al. 2019, 2021). Body illusions provide a useful tool to systematically investigate how the brain integrates sensory inputs from different modalities to create a coherent representation of the body (Blanke and Metzinger 2009; Yau et al. 2015). The rubber hand illusion is the most well‐established paradigm for this purpose (Botvinick and Cohen 1998). In this paradigm, synchronous tactile stimulation of a visible rubber hand and the subject's own hand, which is hidden out of sight, produces a feeling of ownership of the rubber hand.

Various electroencephalography (EEG) studies have investigated spectral power associated with multisensory information processing and MSI during body illusions. In addition to the rubber hand illusion (Faivre et al. 2017; Rao and Kayser 2017; Sciortino and Kayser 2022; Shibuya et al. 2018, 2019, 2021), visuomotor illusions (Li et al. 2023), and virtual reality environments (Evans and Blanke 2013; Lenggenhager et al. 2011) were applied to experimentally manipulate body ownership awareness. These studies revealed reductions in alpha and beta activity over frontal, premotor, somatosensory, posterior parietal, and occipital cortical regions during the illusions, which were related to reduced body localization accuracy and the perception of external objects as being part of one's own body (Lenggenhager et al. 2011; Shibuya et al. 2018, 2019, 2021). Regarding neuroanatomical correlates of the involved processes, the premotor and posterior parietal cortices are considered multisensory areas, as they are composed of heteromodal neurons (Graziano 2001) and respond to different types of sensory stimuli (Bremmer et al. 2001). Therefore, the activation of these areas during illusory states may play a crucial role in the integration of external objects into the body scheme (Ehrsson et al. 2005; Ehrsson and Chancel 2019; Guterstam et al. 2013; Limanowski and Blankenburg 2016). In contrast, the somatosensory and occipital cortices are commonly associated with unisensory processing of tactile and visual stimuli, respectively. Reductions of alpha activity in these areas during body illusions may reflect altered processing demands in the tactile and visual systems in response to conflicting stimuli (Sciortino and Kayser 2022; Shibuya et al. 2018, 2019, 2021).

In addition to studies examining spectral power, previous research has also investigated functional connectivity to examine how interactions between distributed brain regions contribute to the illusory experience, providing complementary information beyond local changes in alpha‐ and beta‐band power. Previous studies support the idea that body illusions emerge from interactions between unisensory and multisensory processing systems. Compared to control conditions, body illusions have been associated with increased connectivity between occipital, premotor, primary motor, somatosensory, and posterior parietal regions (Faivre et al. 2017; Kanayama et al. 2016; Sakai et al. 2020; Zeller et al. 2016). These increases are typically observed in the hemisphere contralateral to the body part stimulated during illusion induction. Such findings have been interpreted in terms of the influence of bottom‐up unisensory inputs facilitating the integration of the external object (Zeller et al. 2016), and integrative effects of multisensory processing on unisensory systems (Kanayama et al. 2016).

It is important to note that most previous EEG studies on body illusions have examined brain spectral power and functional connectivity during multisensory (visuotactile) stimulation, i.e., tactile stimulation of the subject's body and synchronous visual stimulation of an external object. In contrast, changes in neural processing associated with unisensory stimulation have barely been investigated. Nevertheless, accumulating evidence indicates that activity in sensory cortices can be modulated by stimuli from other modalities even in the absence of direct input. For instance, salient sounds have been shown to activate visual cortical areas and modulate occipital alpha activity without visual stimulation, reflecting automatic cross‐modal interactions between sensory systems (Feng et al. 2017; McDonald et al. 2013). Investigating neural responses to unisensory (i.e., visual or tactile) stimulation is critical for understanding how cross‐modal interactions shape sensory processing in multisensory contexts. In line with this idea, Shibuya et al. (2018, 2019, 2021) recorded EEG activity while subjects observed movements of a fake hand after illusion induction, and reported reductions in alpha‐ and beta‐band power similar to those accompanying multisensory stimulation. Interestingly, power reduction during observation of the movement was also seen in cortical areas underlying tactile processing, which gives rise to the hypothesis that effects of unisensory stimulation during the illusion are not restricted to brain regions primarily associated with the stimulation modality.

The aim of this study was to improve our understanding of unisensory and multisensory mechanisms involved in MSI during a sensory conflict. For this purpose, EEG spectral power and functional connectivity were assessed in the rubber hand illusion paradigm and a control condition. As a novel aspect of the study, EEG recordings were separately obtained during unisensory visual, unisensory tactile, and multisensory visuotactile stimulation. All three stimulation types were administered in both an illusion condition, in which the rubber hand was aligned with the participant's real hand, and a control condition, in which the rubber hand was rotated by 180°. Critically, the main focus of the study was to examine differences between the illusion and control conditions. Based on previous research, we hypothesized that the illusion condition would be associated with reduced alpha and beta power compared to the control condition during visuotactile stimulation (Lenggenhager et al. 2011; Shibuya et al. 2018, 2019, 2021). Extending this prediction and considering evidence for cross‐modal influences during unisensory processing, we expected similar reductions during unisensory visual and tactile stimulation. Specifically, we hypothesized that visual stimulation would be accompanied by reduced alpha and beta band power in tactile regions, and that tactile stimulation would be accompanied by reduced activity in visual regions, reflecting cross‐modal reweighting during the illusion. In terms of functional connectivity, we further hypothesized that the illusion condition would be associated with increased connectivity between unisensory and multisensory regions compared to the control condition (Faivre et al. 2017; Kanayama et al. 2016; Sakai et al. 2020; Zeller et al. 2016). Moreover, we expected that unisensory stimulation would not only enhance connectivity within modality‐specific networks but also between modalities, such that visual stimulation would increase connectivity involving tactile regions and vice versa, consistent with the cross‐modal interactions underlying the illusory experience.

## 2 Methods

### 2.1 Participants

Thirty‐eight students from the University of Granada participated in the study (age: M = 19.89 years, SEM = 0.38 years; 32 females), although data from only 36 of them could finally be analyzed (see section 2.5). Power analysis using G*Power (Erdfelder et al. 2009) indicated that a sample size of 34 would be needed to detect differences between conditions (illusion vs. control) with adequate power (1− β = 0.80). This estimation was based on a medium effect size (Cohen's f = 0.25), an *α* error of 0.05, and an assumed correlation of 0.5 between repeated measures. Exclusion criteria for participation were substance abuse, self‐reported mental health issues, and ongoing medical or psychological treatment. This information was gathered during a semi‐standardized interview. The study protocol was approved by the ethics committee of the University of Granada, and all participants provided their informed consent according to the Declaration of Helsinki.

### 2.2 Questionnaires

Participants completed self‐report questionnaires assessing psychological variables with a potential influence on the rubber hand illusion, including handedness (Edinburgh Handedness Inventory [EHI]; Albayay et al. 2019), body connection (Scale of Body Connection [SBC]; Quezada‐Berumen et al. 2014), and suggestibility (Inventory of Suggestibility [IS]; González Ordi et al. 1999). Table 1 presents the means and standard errors of the SBC, IS, and EHI scale scores. According to the EHI, 29 participants were classified as right‐handed, 1 as left‐handed, and 6 as ambidextrous. Overall, participants showed SBC and IS scores in the middle range, indicating moderate levels of body connection and suggestibility within the sample.

**TABLE 1:** Questionnaire scores of the sample; means (M) and standard errors of the mean (SEM).

	M	SEM
Scale of body connection (SBC)		
Body awareness	2.62	0.08
Bodily dissociation	1.11	0.08
Inventory of suggestibility (IS)		
Fantasize	2.73	0.12
Absorption	2.83	0.09
Emotional involvement	2.40	0.12
Influencing	1.86	0.12
Edinburgh handedness inventory (EHI)	51.08	5.59

To assess the degree to which the rubber hand was integrated into the subjects' body scheme, the Pictographic Assessment Embodiment (PAE; Sutil‐Jiménez et al. 2025) was presented during the experiment. The PAE uses a nine‐point Likert scale in which nine squares are depicted, five of which contain pictograms of avatars. The scale ranges from two separated avatars on the extreme left side to two completely overlapping avatars on the extreme right side, symbolizing increasing levels of embodiment with an artificial limb. The PAE constitutes a single scale, reflecting current feelings of embodiment according to its three core components, i.e., the sense of ownership over the rubber hand, the feeling of control or agency, and the perceived self‐location of the rubber hand (Sutil‐Jiménez et al. 2025).

### 2.3 Apparatus and Stimuli

The participants' hands were tactually stimulated using a ring equipped with a vibrator motor operating at a frequency of 110–130 Hz. The rubber hand received visual stimulation via a ring equipped with a light‐emitting diode (LED). Tactile and visual stimuli were controlled by a computer through a parallel port.

### 2.4 Procedure

Data were collected during individual sessions lasting approximately 100 min. First, the participant was briefed about the experiment and completed an informed consent form, followed by an interview and the administration of the EHI, SBC, and IS. Thereafter, the participant was escorted to a quiet and dimly illuminated room, where they sat down in a comfortable chair in front of an opaque box. The EEG cap was then fitted, and the participant received the instructions for the experiment.

**FIGURE 1:**
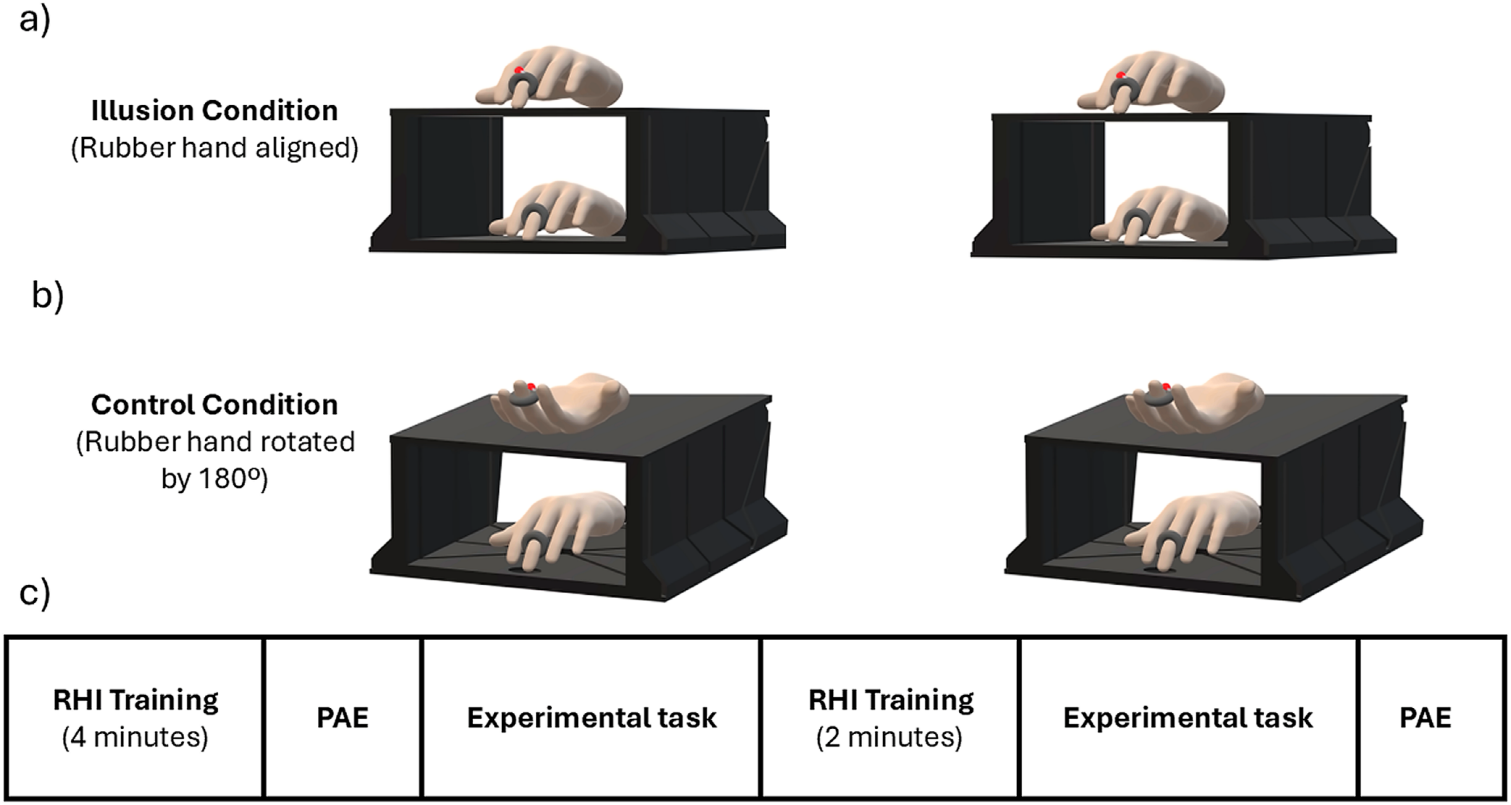
Experimental setup and procedure; (a) rubber hand illusion condition where both hands are aligned and positioned the same way; (b) control condition where the rubber hand is rotated by 180° respect to the real hand; (c) timeline of the experiment (one block); RHI, rubber hand illusion; PAE, pictographic assessment embodiment.

The experimental setup was based on previous studies applying the rubber hand illusion (Botvinick and Cohen 1998; Zeller et al. 2015). The participant was asked to close their eyes and place their left hand, palm down, inside the opaque box. The investigator positioned a rubber hand approximately 10 cm above the subject's hand. This arrangement concealed the real hand, leaving only the rubber hand visible (Figure 1). A black cape covered the gap between the trunk of the rubber hand and the participant's body. Once the setup was complete, the participant was allowed to open their eyes.

The experimental procedure included two counterbalanced blocks corresponding to the illusion and control conditions. The sequence of events within each block was identical for both conditions; the only difference was the position of the rubber hand (see Figure 1a,b). While in the illusion condition, the rubber hand and the subject's hand were aligned in the same position, whereas in the control condition, the rubber hand was rotated by 180° with respect to the real hand (Riemer et al. 2019). Both conditions involved synchronous stimulation of both hands, involving tactile stimulation of the real hand and visual stimulation (i.e., LED illumination) of the rubber hand. In each block, subjects completed the same experimental tasks (i.e., illusion or control) twice. Both task executions were preceded by training periods. After the first training period and the second experimental task, the PAE was presented (see Figure 1c).

#### 2.4.1 Training

Before starting the training, the researcher placed the rings on their own index fingers (vibrotactile ring on the right index finger and LED ring on the left index finger). Thereafter, the researcher synchronously touched the participant's left hand with the vibrotactile ring and the rubber hand with the LED ring, and the participant was instructed to focus their attention on the rubber hand. All fingers of both hands received stimulation. The first and second training sessions of each block lasted 4 and 2 min, respectively. After the training, the researcher placed the vibrotactile ring on the participant's middle finger and the LED ring on the rubber hand's middle finger.

#### 2.4.2 Experimental Task

The two experimental tasks in each block included a 2‐minute adaptation period followed by 20 visual, 20 tactile, and 20 visuotactile stimulation trials, resulting in 60 trials per experimental task. As each block included two task repetitions, this yielded a total of 120 trials per block, corresponding to 40 trials per stimulation type (visual, tactile, and visuotactile). Each trial lasted for 4 s, with intertrial intervals ranging between 2 and 4 s. The order of trials was pseudorandomized within the experimental tasks. The three types of trials were as follows: (i) visual, where the red light illuminated the rubber hand without stimulation of the subject's hand; (ii) tactile, where the ring vibrated on the real hand without illumination of the rubber hand; and (iii) visuotactile, involving a match between the red light on the rubber hand and the vibrotactile stimulation applied to the subject's hand.

### 2.5 EEG Data Acquisition and Processing

The EEG was recorded from Ag/AgCl electrodes with a 32‐channel A.N.T. TMSi Refa8 device (Advanced Neuro Technology, Enschede, the Netherlands). The EEG electrodes were positioned according to the 10/20 montage system; moreover, the electro‐oculogram (EOG) was obtained by placing one electrode above and another one below the left eye. All EEG channels were referenced to the average of the electrodes; electrode impedances were maintained below 10 kΩ. Recordings were digitized at a sampling rate of 1024 Hz and online bandpass filtered between 0 and 70 Hz.

The EEG data were processed offline using the MATLAB toolbox EEGLAB (Delorme & Makeig, 2004). Initially, a 0.1‐Hz high‐pass filter was applied, and eye movement and blink artifacts were corrected using independent component analysis (Jung et al. 2000). The EEG data were segmented into epochs of 2200 ms duration (−200 ms to 2000 ms relative to stimulus onset), with baseline correction applied from −200 ms to 0 ms. The next step involved automatic detection of trials in which the signal of any EEG channel exceeded ±100 μV and/or had an average global difference (i.e., maximum minus minimum value) exceeding ±3 standard deviations of the mean. If an epoch contained three or fewer electrodes with artifacts detected based on this procedure, the signals of these electrodes were interpolated from the signals of adjacent electrodes. Epochs containing more than three electrodes with artifacts were excluded. A minimum of 30 trials per participant and condition was required for power and functional connectivity analyses. Data from two participants had to be discarded due to this criterion. On average, 35 trials per participant were included in further analysis (minimum 30 trials, maximum 38 trials).

### 2.6 Spectral Power Analysis

Spectral density power was estimated for the 8.5–23‐Hz range across all channels with 0.5‐Hz resolution using the Welch method (weighted overlapped segment averaging) (Jwo et al. 2021). The power data were averaged for two frequency bands, i.e., alpha (8.5–13 Hz) and beta (15–23 Hz). Previous studies on body illusions have revealed significant effects for frontocentral, central, parietal, and occipital electrodes (Evans and Blanke 2013; Faivre et al. 2017; Rao and Kayser 2017; Sciortino and Kayser 2022; Shibuya et al. 2021). Therefore, EEG channels were grouped into eight regions, i.e., left frontocentral (FC1 and FC5), right frontocentral (FC2 and FC6), left central (C3), right central (C4), left parietal (P3 and P7), right parietal (P4 and P8), left occipital (O1), and right occipital (O2). The average power in the two frequency bands was computed for each region.

### 2.7 Functional Connectivity

Functional connectivity was assessed using coherence, a frequency‐specific measure of phase synchronization between EEG signals recorded at different electrodes. This metric reflects inter‐regional coupling based on the consistency of phase relationships over time, rather than cross‐frequency interactions. To reduce the influence of volume conduction, we computed the imaginary part of coherence (IC; Nolte et al. 2004), which selectively captures non‐instantaneous interactions between signals and is therefore less sensitive to spurious coupling arising from shared sources. IC was calculated for each epoch and for the alpha and beta frequency bands between all pairs of electrodes.

**FIGURE 2:**
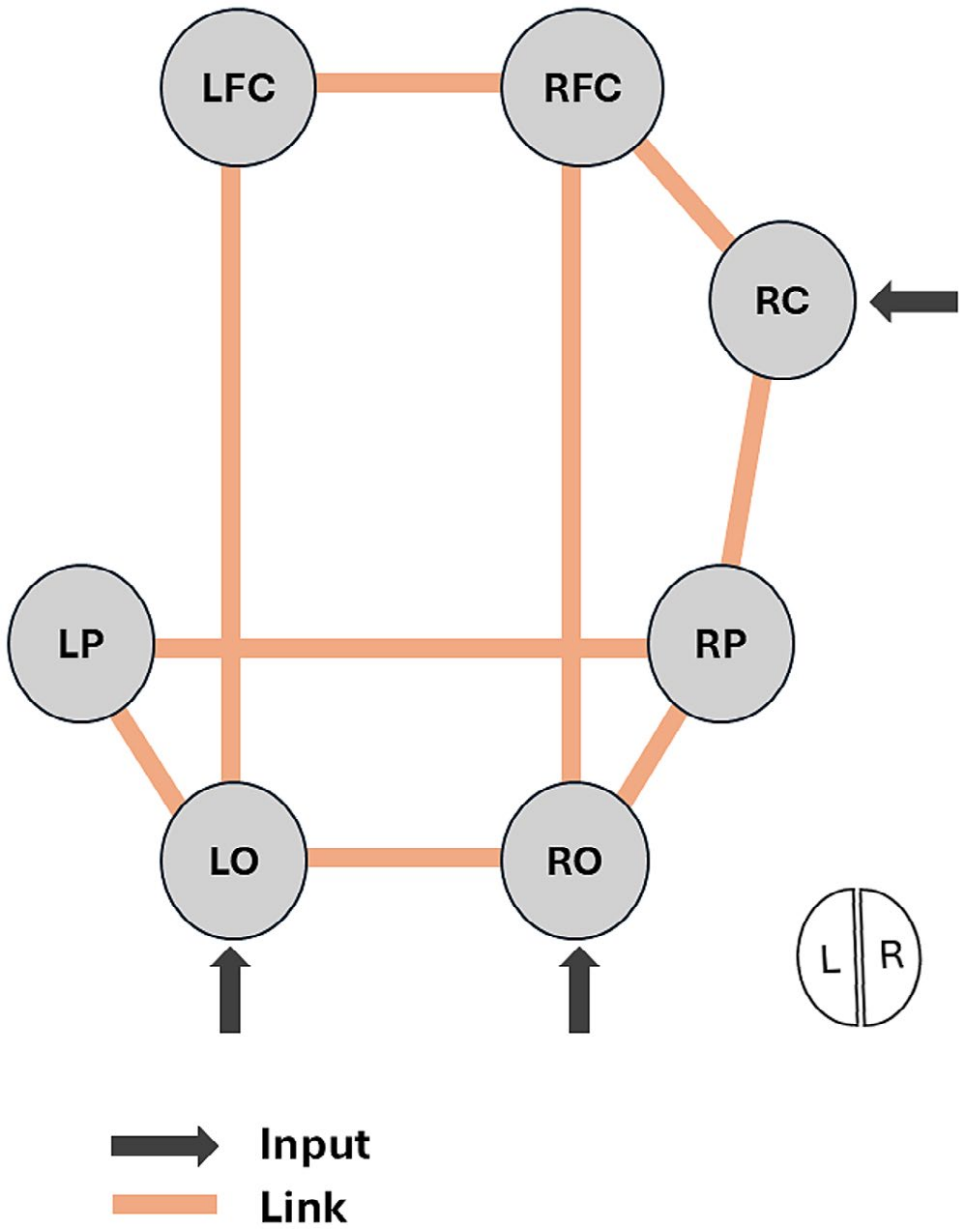
Connectivity scheme for the investigated links between regions of interest (LFC, left frontocentral; RFC, right frontocentral; LC, left central; RC, right central; LP, left parietal; RP, right parietal; LO, left occipital; RO, right occipital).

A region‐of‐interest (ROI) approach was adopted following Zeller et al. (2016), both to provide an unbiased analytical framework and to facilitate comparison with previous studies. The selected connections were guided by our hypotheses (see Introduction) and are illustrated in Figure 2. Occipital and central regions were considered proxies for unisensory (visual and tactile) processing, whereas frontocentral and parietal regions were associated with multisensory integration. Given the contralateral organization of the somatosensory system and the stimulation of the left hand, only connections involving the right central region (corresponding to the right somatosensory cortex) and multisensory areas were included. In contrast, connections between occipital regions in both hemispheres and multisensory areas were examined, as visual input is processed bilaterally.

### 2.8 Statistical Analysis

Statistical analyses were performed with SPSS v. 28 (IBM Corp 2021). The PAE scale was analyzed using repeated‐measures ANOVA with the within‐subjects factors CONDITION (illusion vs. control) and TIME (after first training vs. after second experimental task).

Analyses of spectral power and functional connectivity were performed by means of three‐way repeated‐measures ANOVAs. For alpha and beta power, 2 × 3 × 8 ANOVAs were computed with CONDITION (illusion vs. control), TRIAL TYPE (visuotactile vs. visual vs. tactile), and REGION (left frontocentral vs. right frontocentral vs. left central vs. right central vs. left parietal vs. right parietal vs. left occipital vs. right occipital) as within‐subjects factors. For connectivity, 2 × 3 × 9 ANOVAs were used with CONDITION (illusion vs. control), TRIAL TYPE (visuotactile vs. visual vs. tactile), and LINK (left frontocentral–right frontocentral vs. left frontocentral–left occipital vs. right frontocentral–right central vs. right frontocentral–right occipital vs. right central–right parietal vs. left parietal–right parietal vs. left parietal–left occipital vs. right parietal–right occipital vs. left occipital–right occipital) as within‐subjects factors. Separate models pertaining to connectivity for alpha and beta power were computed.

For ANOVAs, the Greenhouse–Geisser correction (epsilon) was applied when the sphericity assumption was violated. Alpha level was set at 0.05. η_p_^2^ is presented as the effect size measure. In post hoc pairwise mean comparisons (*t*‐tests for dependent samples), Bonferroni corrections were made when needed.

Pearson correlations were conducted to examine the relationship between EEG measures and the perceived strength of the illusory state. Difference scores (illusion–control) were computed for PAE scores obtained after the first RHI induction (PAE1), reflecting the subjective experience of embodiment, as well as for EEG power values averaged across ROIs for each stimulation type (visual, tactile, and visuotactile). These indices were then correlated to assess whether individual variability in illusion‐related EEG modulation was associated with the strength of the illusion.

## 3 Results

### 3.1 Subjective Reports

**FIGURE 3:**
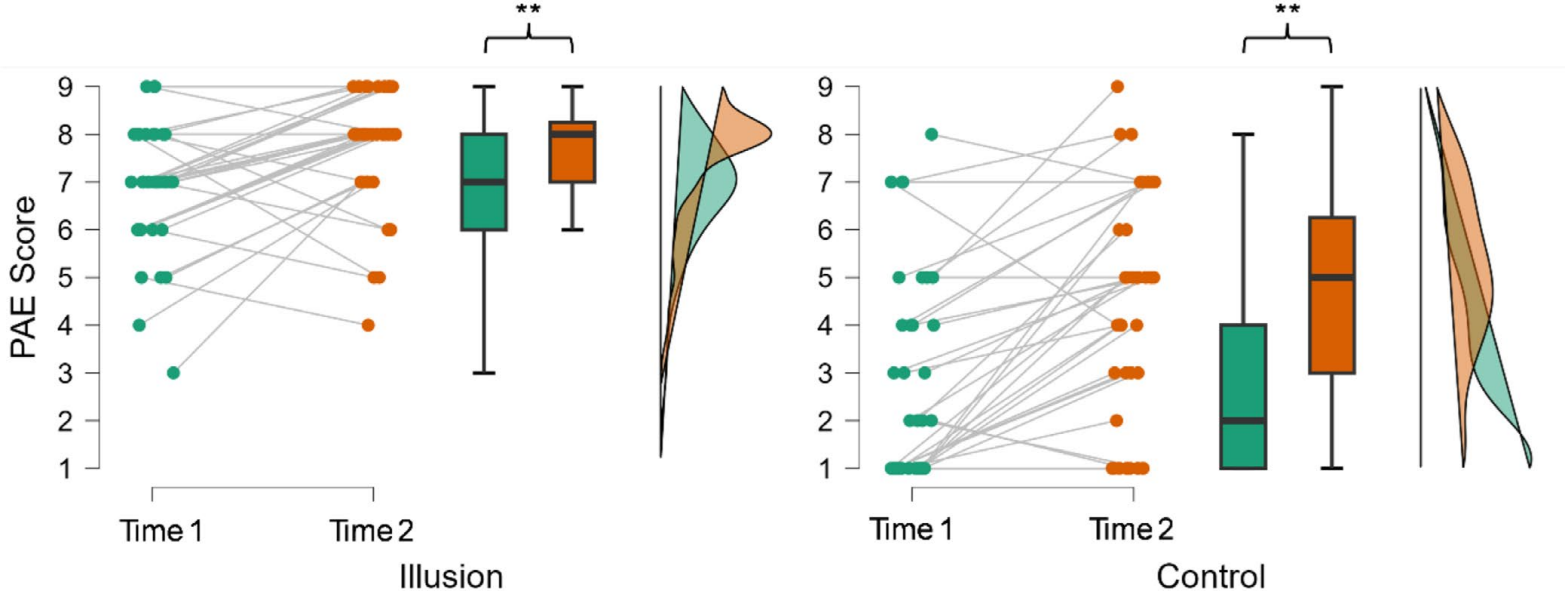
Raincloud and boxplots for the PAE score in the illusion and control conditions after the training (Time 1) and after the second experimental task (Time 2). Box plots indicate distribution of the data with means and standard errors of the mean; ** for *p* < 0.01 regarding the difference between both measurements (Time 1 vs. Time 2).

Figure 3 displays means and standard errors of the mean of the PAE scale scores. The ANOVA for this score revealed main effects of CONDITION (F [1, 35] = 146.190, *p* < 0.001, η_ρ_^2^ = 0.807) and TIME (F [1, 35] = 30.155, *p* < 0.001, η_ρ_^2^ = 0.463). PAE scores were higher for the illusion condition (M = 7.292 ± 0.179) than the control condition (M = 3.597 ± 0.342), and higher after the second experimental task (M = 6.083 ± 0.252) than after the first training (M = 4.806 ± 0.249). Moreover, a CONDITION × TIME interaction arose (F [1, 35] = 6.481, *p* = 0.015, η_ρ_^2^ = 0.156). Pairwise comparisons indicated that PAE scores increased between the two measurements in the illusion (*p* < 0.001) and control conditions (all *p*s < 0.001).

### 3.2 Spectral Density Power

#### 3.2.1 Alpha

**FIGURE 4:**
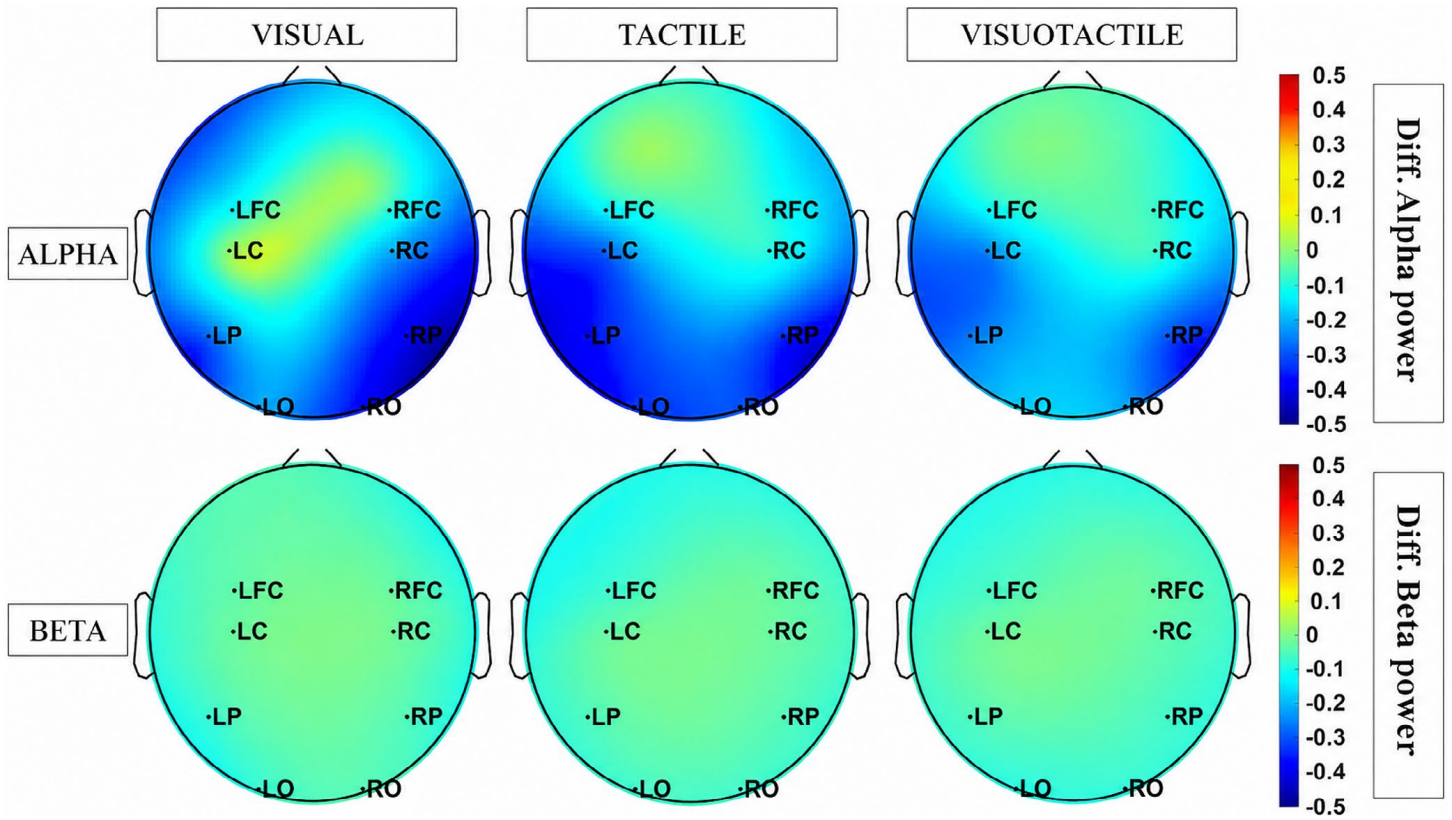
Topographical distribution of the differences between the illusion and control conditions (illusion—control) in alpha and beta power across stimulation types.

The means and standard errors of alpha power for all conditions, trial types, and scalp regions are presented in Table 2, and the ANOVA results for alpha power are shown in Table 3. Consistent with our main hypothesis, alpha power was significantly lower in the illusion condition compared to the control condition (*p* < 0.001). This reduction was observed across stimulation types and scalp regions, as illustrated in Figure 4.

**TABLE 2:** Means and standard errors (in parentheses) of alpha and beta power for all conditions, trial types, and regions.

Trial type	Region	Alpha power (μV^2^/Hz)		Beta power (μV^2^/Hz)	
		Illusion condition	Control condition	Illusion condition	Control condition
Visual stimulation	LFC	**0.676 (0.064)**	**0.798 (0.083)**	**0.361 (0.035)**	**0.435 (0.043)**
	RFC	**0.626 (0.065)**	**0.703 (0.082)**	0.300 (0.030)	0.341 (0.039)
	LC	1.499 (0.274)	1.513 (0.257)	**0.326 (0.039)**	**0.364 (0.044)**
	RC	**0.938 (0.151)**	**1.162 (0.182)**	**0.271 (0.029)**	**0.314 (0.037)**
	LP	**1.529 (0.194)**	**1.848 (0.293)**	**0.503 (0.053)**	**0.565 (0.062)**
	RP	**1.354 (0.161)**	**1.848 (0.277)**	**0.460 (0.055)**	**0.532 (0.071)**
	LO	**1.625 (0.222)**	**1.907 (0.264)**	**0.631 (0.061)**	**0.708 (0.062)**
	RO	**1.560 (0.200)**	**2.028 (0.288)**	0.610 (0.058)	0.688 (0.066)
Tactile stimulation	LFC	**0.580 (0.057)**	**0.693 (0.070)**	**0.351 (0.034)**	**0.418 (0.039)**
	RFC	**0.462 (0.047)**	**0.571 (0.055)**	**0.277 (0.031)**	**0.332 (0.041)**
	LC	**0.799 (0.118)**	**1.100 (0.187)**	**0.276 (0.033)**	**0.332 (0.040)**
	RC	**0.377 (0.042)**	**0.484 (0.054)**	**0.205 (0.024)**	**0.251 (0.031)**
	LP	**1.384 (0.215)**	**1.814 (0.273)**	**0.499 (0.058)**	**0.591 (0.078)**
	RP	**1.291 (0.209)**	**1.678 (0.281)**	**0.451 (0.059)**	**0.549 (0.083)**
	LO	**1.727 (0.263)**	**2.149 (0.320)**	**0.633 (0.060)**	**0.747 (0.068)**
	RO	**1.710 (0.285)**	**2.053 (0.307)**	**0.635 (0.062)**	**0.721 (0.076)**
Visuotactile stimulation	LFC	**0.560 (0.047)**	**0.669 (0.067)**	**0.350 (0.036)**	**0.410 (0.041)**
	RFC	**0.458 (0.039)**	**0.539 (0.054)**	0.279 (0.031)	0.316 (0.038)
	LC	**0.785 (0.100)**	**1.024 (0.171)**	0.281 (0.036)	0.310 (0.037)
	RC	**0.374 (0.035)**	**0.459 (0.048)**	**0.198 (0.023)**	**0.242 (0.028)**
	LP	**1.271 (0.166)**	**1.529 (0.220)**	0.479 (0.050)	0.534 (0.068)
	RP	**1.102 (0.123)**	**1.405 (0.209)**	**0.423 (0.053)**	**0.496 (0.070)**
	LO	**1.473 (0.203)**	**1.656 (0.204)**	**0.585 (0.050)**	**0.678 (0.063)**
	RO	**1.433 (0.197)**	**1.638 (0.215)**	**0.575 (0.050)**	**0.657 (0.067)**

*Note:* Bold values indicate statistically significant post hoc comparisons (*p* < 0.05).

Abbreviations: LC, left central; LFC, left frontocentral; LO, left occipital; LP, left parietal; RC, right central; RFC, right frontocentral; RO, right occipital; RP, right parietal.

**TABLE 3:** Results of the ANOVA for alpha power with the factors CONDITION, TRIAL TYPE and REGION.

	F	df	*p*	η_ρ_^2^
CONDITION	18.718	1, 35	< 0.001	0.348
TRIAL TYPE	15.160	2, 70	< 0.001	0.302
REGION	18.930	7, 245	< 0.001	0.351
CONDITION × TRIAL TYPE	1.192	2, 70	0.301	0.033
CONDITION × REGION	5.395	7, 245	0.003	0.134
TRIAL TYPE × REGION	8.589	14, 490	< 0.001	0.197
CONDITION × TRIAL TYPE × REGION	2.647	14, 490	0.042	0.070

Post hoc comparisons revealed that alpha power was significantly lower in the illusion condition than in the control condition across all regions during tactile and visuotactile stimulation (all *p*s < 0.05). A similar pattern was observed during visual stimulation, with significant differences in all regions except the left central region. Importantly, these effects were not restricted to modality‐specific regions. During visual stimulation, reduced alpha power in the illusion condition was specifically observed in central regions contralateral to the stimulated hand, typically associated with tactile processing, suggesting a cross‐modal influence of visual input on somatosensory areas. Conversely, during tactile stimulation, alpha reductions extended to occipital regions. This indicates enhanced visual processing despite the absence of visual input. These cross‐modal patterns support the idea that the illusion modulates unisensory processing beyond modality‐specific pathways, consistent with a redistribution of sensory weighting across modalities during the illusory state.

Alpha power also varied as a function of trial type (all *p*s < 0.05), being highest during visual stimulation, followed by tactile and visuotactile stimulation. In the control condition, visuotactile stimulation was associated with lower alpha power than visual and tactile stimulation in occipital regions, and lower than tactile stimulation in parietal regions (all *p*s < 0.05). These differences were not observed in the illusion condition, suggesting reduced differentiation between unisensory and multisensory processing once the illusion was established. Differences between trial types in frontocentral and central regions were comparable across conditions.

Pearson correlations were computed between condition‐related differences in alpha power (illusion–control) and individual differences in subjective illusion strength (PAE), separately for each stimulation type. No significant associations were observed (visual: *r* = −0.118, *p* = 0.494; tactile: *r* = −0.041, *p* = 0.814; visuotactile: *r* = −0.058, *p* = 0.735), suggesting that the magnitude of alpha modulation was not directly related to individual variability in the subjective strength of the illusion. In contrast, alpha power differences were strongly correlated across stimulation types (all *p*s < 0.001), indicating a consistent pattern of neural modulation across unisensory and multisensory stimuli.

#### 3.2.2 Beta

Table 4 presents the ANOVA results for beta power, and descriptive statistics are reported in Table 2. Beta power was significantly lower in the illusion condition compared to the control condition (*p* < 0.001), indicating a general reduction associated with the illusory state. In contrast to the alpha band, beta‐band activity did not show clear region‐specific cross‐modal patterns, suggesting that these reductions may reflect a more global modulation rather than spatially specific cross‐modal reweighting. This pattern is illustrated in Figure 4, which displays the topographical distribution of the differences between the illusion and control conditions (illusion—control) across stimulation types.

**TABLE 4:** Results of the ANOVA for beta power with the factors condition, trial type and region.

	F	df	*p*	η_ρ_^2^
CONDITION	15.861	1, 35	< 0.001	0.312
TRIAL TYPE	9.144	2, 70	< 0.001	0.207
REGION	29.288	7, 245	< 0.001	0.456
CONDITION × TRIAL TYPE	1.363	2, 70	0.263	0.037
CONDITION × REGION	1.192	7, 245	0.316	0.033
TRIAL TYPE × REGION	6.131	14, 490	< 0.001	0.149
CONDITION TRIAL TYPE × REGION	0.734	14, 490	0.603	0.021

As expected, beta power also varied as a function of trial type (all *p*s < 0.01), being lower during visuotactile stimulation than during visual and tactile stimulation. A TRIAL TYPE × REGION interaction further indicated that these differences varied across scalp regions.

Pearson correlations between condition‐related differences in beta power (illusion–control) and subjective illusion strength (PAE) revealed a significant negative correlation during visual stimulation (*r* = −0.371, *p* = 0.026), indicating that participants with stronger illusion experiences showed greater beta power reductions. No significant correlations were observed for tactile (*p* = 0.147) or visuotactile stimulation (*p* = 0.067), although the latter showed a non‐significant trend in the same direction.

### 3.3 Functional Connectivity

#### 3.3.1 Alpha

The ANOVA results for alpha‐band IC are presented in Table 5. No main effects or interactions involving CONDITION were observed, indicating that alpha‐band connectivity was not modulated by the illusion.

**TABLE 5:** Results of the ANOVA for functional connectivity in the alpha band with the factors CONDITION, TRIAL TYPE and REGION.

	F	Df	*p*	η_ρ_^2^
CONDITION	0.796	1, 35	0.379	0.022
TRIAL TYPE	0.271	2, 70	0.763	0.008
LINK	241.146	8, 280	< 0.001	0.873
CONDITION × TRIAL TYPE	0.047	2, 70	0.952	0.001
CONDITION × LINK	0.237	8, 280	0.962	0.007
TRIAL TYPE × LINK	2.755	16, 560	0.004	0.073
CONDITION × TRIAL TYPE × LINK	0.962	16, 560	0.472	0.027

#### 3.3.2 Beta

**FIGURE 5:**
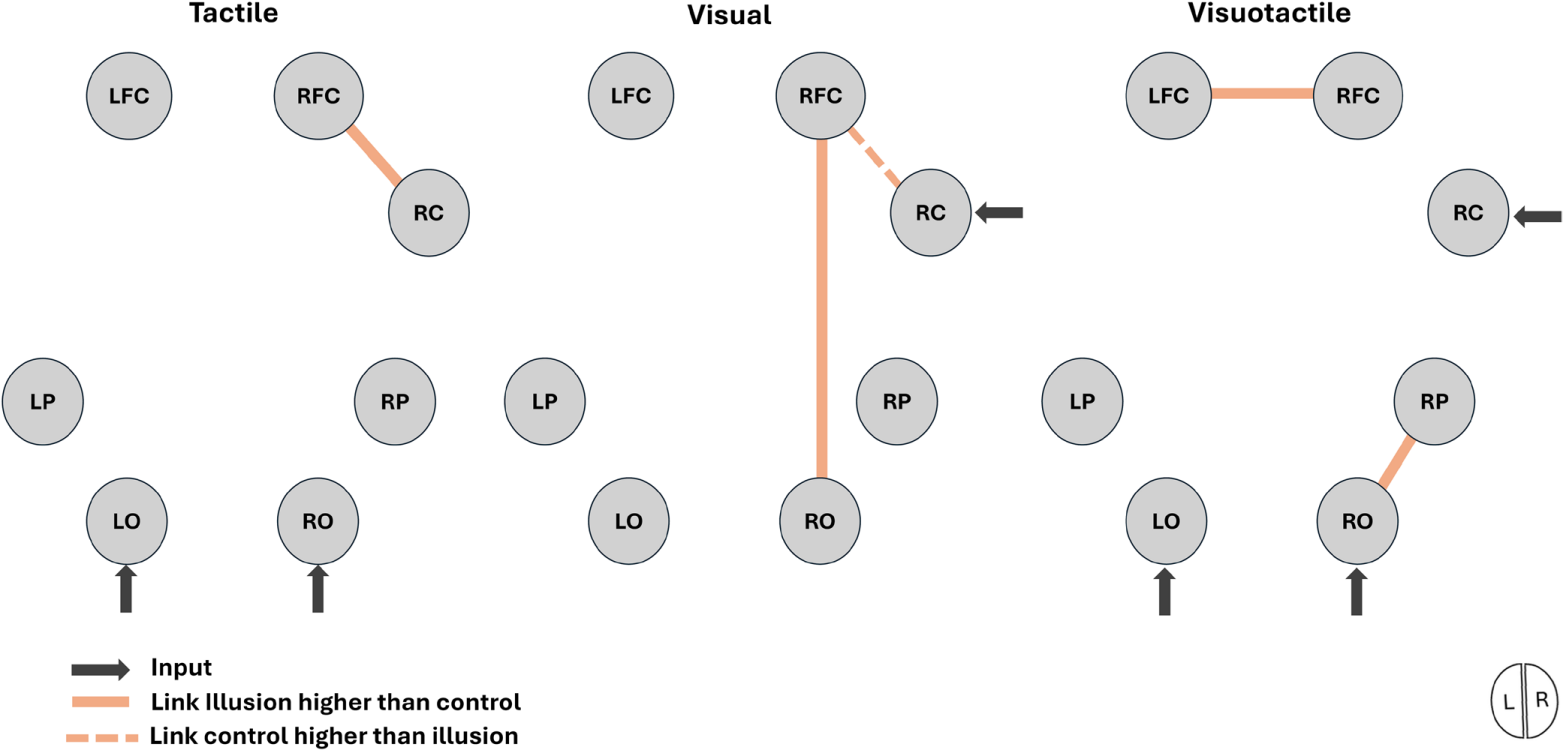
Beta‐band connectivity results displaying the comparison between conditions (illusion vs. control) for the three trial types (visual, tactile and visuotactile). Brown lines represent links with significant differences in IC between the illusion and control conditions. A solid brown line indicates that IC between the corresponding regions is greater in the illusion condition than in the control condition; a dashed brown line indicates that IC between the corresponding regions is greater in the control condition than in the illusion condition. Black arrows represent visual and tactile external inputs. LFC, left frontocentral; RFC, right frontocentral; LC, left central; RC, right central; LP, left parietal; RP, right parietal; LO, left occipital; RO, right occipital.

In contrast, beta‐band connectivity showed a significant effect of CONDITION (Table 6), with overall higher IC values in the illusion condition compared to the control condition (*p* < 0.05), suggesting enhanced functional coupling during the illusory state. This effect is illustrated in Figure 5.

**TABLE 6:** Results of the ANOVA for functional connectivity in the beta band with the factors CONDITION, TRIAL TYPE and REGION.

	F	df	*p*	η_ρ_^2^
CONDITION	9.998	1, 35	0.003	0.222
TRIAL TYPE	0.994	2, 70	0.375	0.028
LINK	227.166	8, 280	< 0.001	0.866
CONDITION × TRIAL TYPE	2.068	2, 70	0.134	0.056
CONDITION × LINK	0.320	8, 280	0.895	0.009
TRIAL TYPE × LINK	2.048	16, 560	0.009	0.055
CONDITION × TRIAL TYPE × LINK	2.026	16, 560	0.031	0.055

Follow‐up analyses revealed that this modulation was not uniform across connections but depended on trial type and specific inter‐regional links, as reflected in a CONDITION × TRIAL TYPE × LINK interaction. During visual stimulation, increased beta‐band connectivity in the illusion condition was observed between frontocentral and central regions, consistent with enhanced coupling between multisensory and somatosensory areas in the absence of tactile input.

During tactile stimulation, a mixed pattern emerged, with increased connectivity in the illusion condition for frontocentral–occipital links, alongside reduced connectivity for frontocentral–central links, suggesting a redistribution of coupling across sensory systems.

During visuotactile stimulation, greater beta‐band connectivity in the illusion condition was observed in frontocentral–frontocentral and parietal–occipital links, indicating strengthened interactions within multisensory networks and between multisensory and visual regions.

Overall, these findings indicate that the illusion is associated with a reconfiguration of functional connectivity patterns, particularly involving interactions between unisensory and multisensory regions, rather than a uniform increase across the network.

## 4 Discussion

This study investigated the neural mechanisms underlying unisensory and multisensory processing during a state of sensory conflict induced by the rubber hand illusion. Behavioral results confirmed successful illusion induction, as reflected by higher PAE scores in the illusion compared to the control condition. Consistent with our main hypothesis, both alpha and beta power were reduced in the illusion condition across stimulation types. The reductions replicate the results of previous studies (Faivre et al. 2017; Lenggenhager et al. 2011; Rao and Kayser 2017; Sciortino and Kayser 2022; Shibuya et al. 2018, 2019, 2021), supporting the idea that decreased alpha and beta power is a hallmark of the illusory state. Notably, similar reductions were observed during purely visual and tactile trials, suggesting that the illusion alters unisensory processing beyond modality‐specific pathways. This pattern was particularly evident in the cross‐modal effects observed in the alpha band, where visual stimulation modulated activity in somatosensory regions and tactile stimulation influenced occipital areas. Together, the cross‐modal effects support the idea that embodiment is associated with a redistribution of sensory weighting across modalities. These cross‐modal effects observed during unisensory stimulation can be interpreted within complementary theoretical frameworks. They may reflect sustained top‐down predictions associated with the embodied state, whereby the brain maintains an internal model of the rubber hand as part of the body, leading to modality‐independent modulation of sensory cortices (Faivre et al. 2017; Sciortino and Kayser 2022). Alternatively, these effects may be explained by attentional mechanisms, such that once ownership is established, attention is preferentially allocated to the rubber hand, enhancing processing in visual and sensorimotor regions regardless of the incoming sensory modality (Shibuya et al. 2021). More broadly, these findings may also be interpreted within frameworks of sensory priors and causal inference in multisensory perception. The observation that unisensory stimulation evokes multisensory‐like neural patterns in the illusion condition is consistent with the idea that, once the rubber hand is incorporated into the body representation, prior expectations about its ownership bias the inference that visual and somatosensory signals originate from a common cause (Chancel et al. 2022; Ehrsson and Chancel 2019; Samad et al. 2015). In this context, unisensory inputs may be interpreted in light of an already established multisensory model of the body. Although the present design does not allow direct testing of these theoretical accounts, the widespread nature of the observed modulations across stimulation types is consistent with a contribution of predictive, attentional, and causal inference mechanisms to the maintenance of the illusory state. Correlational analyses further showed that beta power reductions during visual stimulation were associated with the subjective strength of the illusion, whereas alpha modulation did not show such a relationship, suggesting partially dissociable roles of these frequency bands in indexing the illusory state. Extending these findings, connectivity analyses revealed changes at the network level. While spectral power reflects local changes in cortical activity, connectivity captures interactions between distributed sensory and multisensory regions. In this context, beta‐band connectivity was enhanced in the illusion condition, although this effect depended on stimulation type and specific inter‐regional links. These changes were primarily observed between unisensory and multisensory regions (Faivre et al. 2017; Kanayama et al. 2016; Sakai et al. 2020; Zeller et al. 2016), pointing to a reconfiguration of functional coupling across sensory processing networks.

### 4.1 Alpha Power

Alpha power was consistently reduced in the illusion condition compared to the control condition across stimulation types and scalp regions. According to neuroimaging studies, changes in activity within multisensory areas, such as parietal and premotor regions, during body illusions reflect the sensory integration of external objects into the body schema (Ehrsson et al. 2005; Ehrsson and Chancel 2019; Guterstam et al. 2013; Limanowski and Blankenburg 2016). The observed reduction in alpha activity in these regions may reflect increased engagement of multisensory networks supporting the incorporation of the rubber hand into the body representation. Previous research has consistently shown that the illusion increases the involvement of multisensory areas during visuotactile stimulation (Lenggenhager et al. 2011; Rao and Kayser 2017; Sciortino and Kayser 2022). Extending these findings, the present results demonstrate that similar neural modulations are also present during purely visual and tactile stimulation. This reinforces the idea that the illusory state is associated with multisensory integration of the external object, affecting not only visuotactile processing—where multisensory integration is expected—but also unisensory processing.

Considering the crucial role of the occipital cortex in visual processing, reduced occipital alpha power during the rubber hand illusion has previously been linked to increased visual attention to the rubber hand (Shibuya et al. 2019, 2021). Notably, in the present study, occipital alpha activity in the illusion condition was reduced not only during visual and visuotactile stimulation but also during purely tactile stimulation. This pattern suggests increased engagement of visual cortical regions even in the absence of visual input. Moreover, previous studies have shown that visual stimulation of the rubber hand can activate sensorimotor areas typically involved in processing tactile input to the real hand (Shibuya et al. 2018, 2019, 2021). Consistent with this, the present results showed reduced alpha power during visual stimulation at central electrodes over sensorimotor cortical regions. Taken together, these findings suggest that the illusory state increases reciprocal influences between visual and somatosensory systems, consistent with cross‐modal reweighting during embodiment of the rubber hand.

In addition, alpha activity was modulated as a function of multisensory integration demands. In bilateral parietal and occipital regions, visuotactile stimulation was associated with lower alpha power than visual and tactile stimulation in the control condition, whereas this difference was not observed in the illusion condition. These findings indicate that the distinction between unisensory and multisensory processing is reduced once the illusion is established. More generally, the widespread reduction in alpha power across all stimulation types in the illusion condition may reflect a more uniform mode of sensory processing, in which differences between unisensory and multisensory inputs are attenuated. This pattern suggests that the modulation observed in the alpha band could be associated with reduced demands on multisensory integration, possibly reflecting a diminished sensory conflict between visual and tactile signals.

Interestingly, no significant associations were found between alpha power modulation and subjective reports of the illusion as measured by the PAE. While this might suggest that alpha‐band activity does not track individual differences in global embodiment, previous studies have reported correlations between alpha desynchronization and more specific indices of the illusion, such as ownership ratings and proprioceptive drift (Shibuya et al. 2019, 2021). One possible explanation is that alpha power is more closely related to specific sensory components of the illusion, rather than to global measures of embodiment that aggregate multiple dimensions (e.g., ownership, location, and agency). In this sense, alpha‐band modulation may primarily reflect changes in sensory processing and integration, which do not necessarily scale with the overall subjective strength of the illusion.

### 4.2 Beta Power

Beta power was significantly lower in the illusion condition than in the control condition, independent of stimulation type and scalp region, indicating a global modulation of beta‐band activity associated with the illusory state. In addition, beta activity varied as a function of stimulation type, with lower power observed during visuotactile stimulation compared to purely visual and tactile conditions. This pattern is consistent with previous findings and may reflect increased processing demands during multisensory stimulation (Friese et al. 2016; Göschl et al. 2015). However, these differences were observed across both experimental conditions and therefore do not appear to be specific to the illusory state.

Importantly, beta power reductions were significantly associated with subjective reports of illusion strength specifically during visual stimulation. Participants who reported stronger embodiment exhibited greater decreases in beta activity, even in the absence of tactile input. This finding suggests that beta‐band modulation may be closely linked to the subjective experience of the illusion. This interpretation is consistent with previous studies showing both beta‐band suppression during body illusions and its relationship with subjective measures of ownership. For example, Faivre et al. (2017) reported reduced beta power during synchronous visuotactile stimulation, alongside correlations with illusory self‐touch, while Shibuya et al. (2021) found that beta desynchronization was associated with ownership ratings. Together, these findings converge in suggesting that beta power plays a key role in the subjective experience of embodiment during body illusions. Notably, in the present study this relationship emerged during visual stimulation alone, indicating that beta‐band activity may reflect not only specific components of embodiment, but also more global aspects of the embodied experience, even in the absence of concurrent tactile input.

### 4.3 Functional Connectivity

Regarding functional connectivity, beta‐band connectivity for the investigated links was overall higher in the illusion condition than the control condition, indicating enhanced coupling between brain regions during the illusory state. This finding is consistent with previous studies suggesting that body illusions involved increased interactions between unisensory and multisensory brain areas (Faivre et al. 2017; Kanayama et al. 2016; Sakai et al. 2020; Zeller et al. 2016). In the present study, this effect was observed across stimulation types and involved connections between regions associated with unisensory processing (e.g., central and occipital areas) and multisensory integration (e.g., frontocentral and parietal regions), supporting the idea that the illusion emerges from dynamic interactions across distributed sensory networks (Faivre et al. 2017; Kanayama et al. 2016; Zeller et al. 2016).

Although some connectivity patterns varied depending on stimulation type, these effects were not uniform across links, suggesting that the illusion is associated with a reconfiguration of functional connectivity rather than a simple increase in coupling. For instance, during visual stimulation, connectivity between frontocentral and central regions contralateral to the stimulated hand increased in the illusion condition, whereas it decreased during tactile stimulation. It has been established that, following the induction of the rubber hand illusion, somatosensory responses to stimulation of the real hand are reduced, as reflected by decreased activity in the somatosensory cortex (Hornburger et al. 2019; Limanowski 2022; Sakamoto and Ifuku 2021; Zeller et al. 2015). Accordingly, reduced beta‐band connectivity in this link during tactile stimulation may reflect diminished sensorimotor engagement. In contrast, increased connectivity during visual stimulation may indicate enhanced sensorimotor recruitment in the absence of tactile input, consistent with evidence that visual stimulation of the rubber hand can elicit sensorimotor responses during the illusion (Shibuya et al. 2018, 2019, 2021). Taken together, these findings suggest that beta‐band connectivity in this link reflects modality‐dependent changes in sensorimotor processing, consistent with a redistribution of sensory weighting across modalities during the illusion.

Tactile stimulation in the illusion condition was also associated with increased beta‐band connectivity between frontocentral and occipital regions. A similar frontal–occipital coupling has been reported by Zeller et al. (2016), who interpreted it as reflecting interactions between visual and somatosensory processes during the illusion. In the present study, this pattern may indicate enhanced cross‐modal coupling, whereby visual processing of the rubber hand remains engaged even during tactile stimulation of the real hand. This interpretation is consistent with the observed reduction of occipital alpha power during tactile stimulation, suggesting increased visual involvement in the illusory state. Together, these findings support the idea that the illusion is associated with increased interplay between visual and somatosensory systems, contributing to the updating of body representation.

During visuotactile stimulation, increased beta‐band connectivity was also observed in parietal–occipital and bilateral frontocentral links in the illusion condition. While these findings are broadly consistent with the involvement of distributed multisensory networks, they were not specifically predicted and should therefore be interpreted with caution. In particular, modulations in parietal–occipital connectivity have not been consistently reported in previous studies, suggesting that these effects may reflect task‐specific dynamics or additional network‐level adjustments during multisensory stimulation in the illusory state. One possible interpretation of the increased interhemispheric frontocentral connectivity is that it reflects enhanced bilateral premotor engagement during the illusory state. The premotor cortex has been consistently implicated in body ownership and visuomotor integration, and bilateral recruitment may support the integration of visual and somatosensory signals across hemispheres (Peviani et al. 2018; Zeller et al. 2011). Alternatively, this pattern may be related to attentional mechanisms, as the illusion requires sustained attention to the rubber hand and may involve shifts in spatial attention across sensory modalities (Rao and Kayser 2017). In this context, increased interhemispheric coupling could reflect the coordination of attentional resources across hemispheres. Although these interpretations remain speculative, they suggest that interhemispheric interactions may contribute to the maintenance of the illusory state and warrant further investigation.

In contrast, alpha‐band connectivity was not modulated by the illusion, suggesting that connectivity changes may be frequency‐specific. While both alpha and beta power were sensitive to the experimental manipulations, only beta‐band activity showed consistent changes in inter‐regional coupling. This divergence may reflect functional differences between frequency bands, with alpha‐band power primarily indexing local processing and beta‐band connectivity supporting long‐range coordination across sensory networks. Previous EEG studies on multisensory integration have reported connectivity effects in different frequency ranges (e.g., Faivre et al. 2017; Kanayama et al. 2016; Zeller et al. 2016), making direct comparisons difficult. In this context, the present findings suggest that beta‐band connectivity may play a specific role in the dynamic coordination of distributed sensory networks underlying the illusory experience, whereas alpha‐band effects may be more closely related to local changes in cortical excitability.

### 4.4 Future Perspectives and Conclusions

The use of unisensory stimulation within the rubber hand illusion represents a novel approach to the study of multisensory integration, and further work is needed to fully explore its potential and limitations. In the present study, behavioral assessment of embodiment was limited to the PAE, which successfully confirmed the induction of the illusion while providing a measure of global embodiment. However, future studies would benefit from incorporating additional behavioral and psychophysical measures, such as proprioceptive drift (Botvinick and Cohen 1998; Tosi et al. 2023) or two‐alternative forced‐choice tasks (Chancel and Ehrsson 2020), which may provide a more fine‐grained characterization of the illusory experience. In particular, measures such as proprioceptive drift may help dissociate perceptual components of embodiment from more cognitive or subjective aspects, thereby improving the interpretation of the relationship between behavioral and neural indices of the illusion. Recent psychophysical approaches allow modeling how multisensory information is weighted during the emergence of body ownership (Chancel and Ehrsson 2020; Lanfranco et al. 2023, 2024). In this context, such methods may help dissociate the multisensory integration processes that give rise to the illusion from those underlying conscious access to the embodied percept. Combining these approaches with EEG could therefore provide a more precise characterization of the neural mechanisms supporting different components of the illusory experience. In addition, spectral analyses were conducted without explicitly separating periodic and aperiodic components of the power spectrum. Recent methodological advances have highlighted the importance of this distinction for the interpretation of oscillatory activity (Donoghue et al. 2020). Although the observed effects showed frequency‐specific and condition‐dependent patterns, future studies should apply spectral parameterization approaches to further validate the oscillatory nature of these findings. Finally, the use of alternative paradigms, such as virtual reality, may further extend this line of research. Virtual environments have been shown to induce strong embodiment experiences and modulate proprioceptive representations (Lenggenhager et al. 2011), offering a flexible framework to investigate the role of unisensory and multisensory processes in body representation.

In summary, the present study demonstrates that changes in embodiment induced by the rubber hand illusion are reflected in both unisensory and multisensory neural processes. The illusion was associated with widespread reductions in alpha and beta power, alongside increased beta‐band functional connectivity between unisensory and multisensory cortical regions. The reductions in power suggest local changes in cortical activity related to the resolution of sensory conflict and cross‐modal reweighting of unisensory processes following embodiment of the rubber hand (Ehrsson and Chancel 2019; Guterstam et al. 2013; Limanowski and Blankenburg 2016). Consistent with these local effects, connectivity analyses revealed increased interactions between unisensory and multisensory regions, reflecting modality‐dependent changes in sensorimotor and visual processing (Faivre et al. 2017; Kanayama et al. 2016; Zeller et al. 2016). Importantly, the use of unisensory stimulation revealed that these effects are not limited to multisensory contexts, but extend to purely visual and tactile processing. This supports the idea that embodiment is associated with a global reconfiguration of sensory processing, characterized by cross‐modal interactions across sensory modalities.

## Author Contributions

**A. J. Sutil‐Jiménez:** investigation, writing – original draft, writing – review and editing, visualization, data curation, formal analysis, software. **S. Duschek:** writing – review and editing. **G. Alba:** investigation, methodology, writing – review and editing, visualization, formal analysis, software, data curation. **M. A. Muñoz:** conceptualization, funding acquisition, resources, project administration, writing – review and editing, supervision, methodology.

## Funding

This work was supported by the Spanish Ministry of Economy, Industry and Competitiveness (PSI2017‐88388‐C4‐3‐R) and by Regional Ministry of Economy, Knowledge, Enterprise and Universities of Andalusia (B‐SEJ‐028‐UGR18). The Mind, Brain and Behavior Research Center receives funding from grants CEX2023‐001312‐M/AEI/ and UCE‐PP2023‐11/UGR by the University of Granada. The funding sources had no involvement in study design, data collection and analysis, decision to publish, or preparation of the manuscript. The authors declare no competing interests.

## Conflicts of Interest

The authors declare no conflicts of interest.

## Data Availability

The data that support the findings of this study are available from the corresponding author upon reasonable request.
